# Pharmacological inhibition of METTL3 impacts specific haematopoietic lineages

**DOI:** 10.1038/s41375-023-01965-2

**Published:** 2023-07-19

**Authors:** Katherine Sturgess, Eliza Yankova, M. S. Vijayabaskar, Tomoya Isobe, Justyna Rak, Iwo Kucinski, Melania Barile, Natalie A. Webster, Maria Eleftheriou, Rebecca Hannah, Malgorzata Gozdecka, George Vassiliou, Oliver Rausch, Nicola K. Wilson, Berthold Göttgens, Konstantinos Tzelepis

**Affiliations:** 1 Wellcome-MRC Cambridge Stem Cell Institute, University of Cambridge, Cambridge, CB2 0AW UK; 2 Department of Haematology, University of Cambridge, Cambridge, CB2 0AW UK; 3 Milner Therapeutics Institute, University of Cambridge, Puddicombe Way, Cambridge, CB2 0AW UK; 4 Storm Therapeutics Ltd, Babraham Research Campus, Cambridge, CB22 3AT UK; 5 Experimental Cancer Genetics, Wellcome Trust Sanger Institute, Hinxton, Cambridge, CB10 1SA UK

**Keywords:** Haematopoietic stem cells, Translational research, Stem-cell research

## To the Editor:

Recent efforts in understanding the epitranscriptome have shown that a diverse set of modifications to RNA represent a new pervasive mechanism of gene regulation, with roles in stem cell homeostasis and disease. N^6^-methyladenosine (m^6^A) is an evolutionarily conserved RNA modification and one of the most abundant found on polyadenylated RNA [1, 2]. The modification is predominantly deposited on mRNA by the METTL3/METTL14 methyltransferase complex [3, 4]. The majority of the reported phenotypes connected to METTL3/METTL14 function have so far utilised genetic knock-down or knock-out approaches which have been proven fairly pleiotropic, mainly due to the significant negative impact on the general m^6^A complex [3, 4]. Lack of reagents and strategies to selectively block the catalytic activity of METTL3 without affecting any of its other functions and interactions has hindered investigation of catalysis-specific METTL3 activity. We recently showed that pharmacological inhibition of the catalytic activity of METTL3, using the first-in-class small molecule STM2457, is a novel therapeutic strategy against acute myeloid leukaemia (AML) [5]. While no toxicity or long-term effects on normal blood counts were observed after in vivo pharmacological inhibition using STM2457, the potential impact of the isolated catalytic inhibition of METTL3 on normal haematopoiesis remained elusive. To address this, here we utilize a high-resolution single cell RNA sequencing (scRNA-seq) approach to understand: 1) the effect of catalytic inhibition of METTL3 on different lineages within normal haematopoiesis and 2) its specific impact on haematopoietic stem cell fate decisions in vivo.

**Fig. 1:**
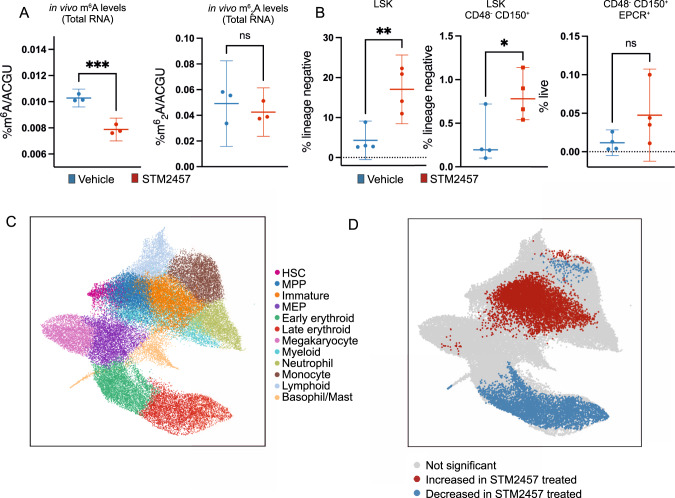
Pharmacological inhibition of METTL3 induces lineage bias in the earliest haematopoietic progenitors. **A** RNA-mass spectrometry quantification of in vivo m^6^A and m^6^_2_A levels on total RNA using bone marrow from treated mice with vehicle or 50 mg/kg STM2457 (mean ± s.d.,*n* = 3). *P* values are indicated as follows: *p < 0.05, **p < 0.01, ***p < 0.005. **B** Frequency of bone marrow LSK, LSK CD48- CD150+ HSCs (as percentage of lineage-negative cells) and CD48- CD150 + EPCR+ HSCs (as percentage of live cells). Values are shown as individual points with mean and SD. *P* values were calculated using independent 2-tailed *t*-test. *P* values are indicated as follows: *p < 0.05, **p < 0.01, ***p < 0.005. **C** UMAP representation of the integrated scRNA-seq dataset containing cells from both STM2457 and vehicle-treated LK bone marrow. Clusters are named according to the predominant cell-type present. Contaminating clusters of mature cell-types and clusters containing <2% of the total dataset were not considered in downstream cluster-based analysis and are not included in this representation (50547 cells shown). **D** Statistically significant cellular abundance changes. *P* values were calculated using an independent 2-tailed *t*-test. Cells with Benjamini-Hochberg corrected *p* values < 0.2 are shown in red if their abundance is increased or blue if depleted in the STM2457-treated bone marrow. LSK lineage negative, Sca1 positive, Kit positive, HSC Haematopoietic stem cell, MPP Multipotent progenitor, MEP Megakaryocyte-erythroid progenitor.

To investigate the above, we initially performed in vivo studies using wild-type CB57BL/6 N mice treated daily with either vehicle or 50 mg/kg of STM2457 over the course of 2 weeks (Fig. S1A). We confirmed effective and selective in vivo catalytic inhibition of METTL3 as total m^6^A modification levels on RNA were significantly reduced after treatment with STM2457 while no changes were detected on m^6^_2_A RNA modification levels (Fig. 1A). Consistent with previous reports using METTL3 knock-out (KO) mouse models [6–8], flow cytometric analysis of the harvested bone marrow from the 2 treated cohorts demonstrated expansion of the haematopoietic stem and progenitor cell (HSPC) compartment of STM2457-treated mice, with increased numbers of lineage- c-kit+ Sca1+ (LSK) and LSK CD150 + CD48- HSCs (Figs. 1B and S1B).

To reveal gene expression changes across the haematopoietic stem/progenitor landscape following pharmacological inhibition of METTL3, we performed scRNA-seq on lineage negative, kit positive (LK) haematopoietic stem and progenitor cells from 3 mice treated with STM2457 and 4 vehicle-treated controls (Table S1). After filtering, 52,424 single-cell transcriptomes were visualised in an integrated universal manifold approximation and projection (UMAP) embedding (Figs. 1C and S1C–G).

Differential gene expression between treated and untreated conditions identified 152 upregulated and 89 downregulated genes (Table S2). Consistent with the previously identified aberrant stabilization of double-stranded RNA molecules following conditional METTL3 KO [8], gene set enrichment analysis identified upregulated inflammatory pathways and cytokine response, while heme biosynthesis was the most significantly downregulated term (Fig. S2A). Taking the inflammatory response to virus signature as a surrogate for successful catalytic inhibition of METTL3, the genes in this signature (GO:0051607) were used to compute a ’defense to virus’ score (Fig. S2B). This was significantly upregulated across all clusters and cell types of the haematopoietic stem and progenitor compartment in STM2457-treated compared to vehicle-treated, indicating that in all cell types, successful inhibition of METTL3 induced a double-stranded RNA response through aberrant transcript stabilization.

To define tissue-level effects of METTL3 inhibition in detail, we performed differential cell abundance testing (Fig. 1D). In contrast to reports using METTL3 KO mouse models [6–8], this analysis demonstrated no significant perturbation in cell numbers in the least-committed progenitors, including HSCs. The discordance between phenotypic and transcriptional HSC abundance likely reflects inflammation-related upregulation of phenotypic markers including Sca1 in the inhibitor-treated bone marrow (Fig. S1C). The most marked difference in cellular abundance was a significant reduction in erythroid progenitors, occurring downstream of megakaryocyte-erythroid progenitors (MEPs), and accompanied by an increase in the abundance of neutrophil progenitors. The lineage-specificity of these findings is in contrast with published reports of scRNA-seq analyses in METTL3 KO mouse models [6], which have demonstrated more widespread depletion in lineage-primed progenitors and mature cell types, potentially due to catalytic-independent effects originating from METTL3 protein loss. The results presented here suggest that pharmacological inhibition of METTL3 induces lineage bias in the earliest haematopoietic progenitors.

**Fig. 2:**
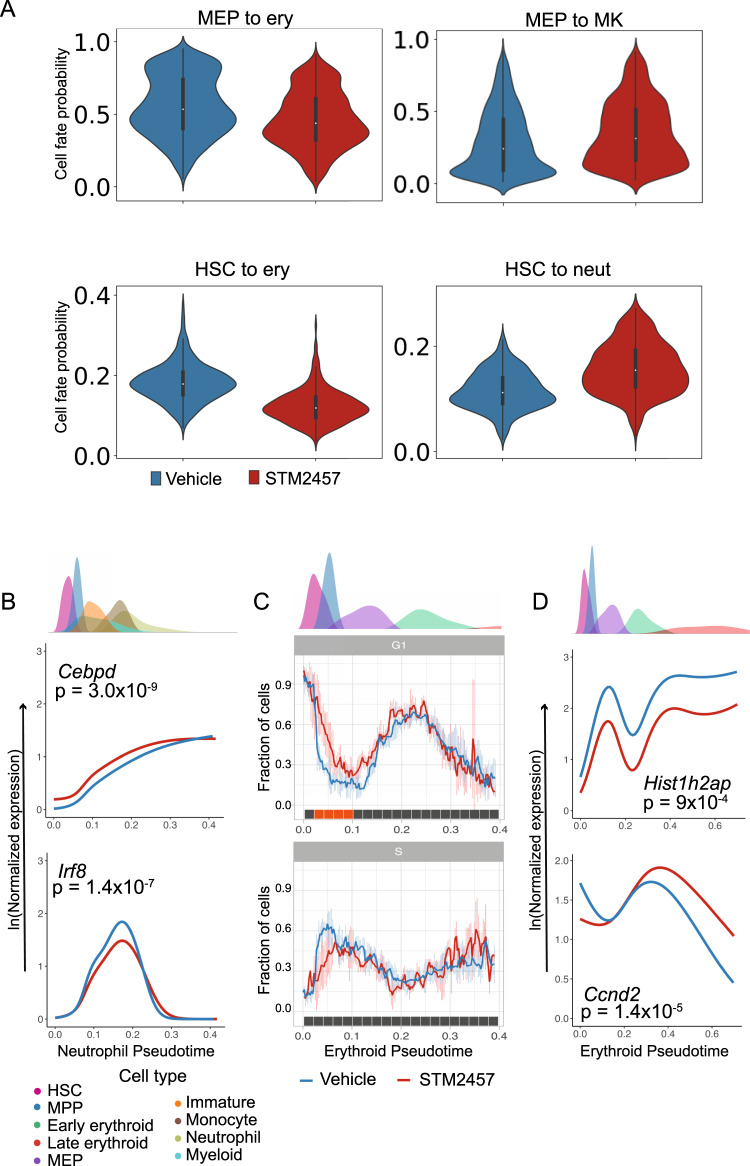
Catalytic inhibition of METTL3 in vivo impacts erythroid differentiation and maturation. **A** Significant difference in cell fate probability in the MEP cluster (top panel): erythroid (left, adj. *p* = 1.65e−52) and megakaryocyte probabilities (right, adj *p* = 2.86e−19). Erythroid probability is significantly reduced in the HSC cluster (bottom panel, left, adj *p* = 9.55e−86), while neutrophil probability is increased (bottom panel, right, adj *p* = 3.26e−62). **B** Differential gene expression dynamics in the neutrophil trajectory. The upper panel shows the density distribution of cell types along pseudotime. The lower panels show gene expression smoothers (line plots) calculated using a generalised additive model for STM2457-treated (red) and vehicle-treated (blue) samples. For *p* value calculation see materials and methods. **C** Dynamic changes in cell cycle along the erythroid trajectory from HSCs to late erythroid progenitors. Upper panel shows the density distribution of cells along erythroid pseudotime. Lower panels present the normalized fraction of cells transcriptomically assigned to G1 or S phase along the early erythroid trajectory. Along a given trajectory, the mean fraction of cells that are in a cell cycle phase for each condition was computed. This was performed along a sliding window with a width of 0.01 and step size of 0.0025. More STM2457-treated HSCs, MPPs and MEPs are in G1 phase, and fewer STM2457-treated progenitors are in S-phase. Cells occupying pseudotime earlier than 0.002, representing the earliest progenitors, including LT-HSCs, show no significant difference in cell cycle phase, while pseudotemporally later cells show a separation between STM2457-treated and vehicle-treated, with a trend towards lower S phase and significantly higher G1 phase in the STM2457-treated samples. Mean (coloured line) of STM2457-treated (*n* = 3) and vehicle-treated (*n* = 4) samples are shown. The error bars indicate the standard deviation of the fraction of cells across the experiments within a condition. The coloured bar aligned with the x-axis denotes significant differences between STM2457 and vehicle-treated cells (adjusted *p* < 0.05 orange, adjusted *p* > 0.05 grey). *P* values were calculated using a two-tailed *t*-test with BH correction. **D** Differential gene expression dynamics in erythroid trajectory (see **B** for method).

To ascertain the level at which lineage bias originates, we performed cell fate probability analysis with CellRank [9] which estimates the probability of a cell’s commitment to each of the seven major haematopoietic lineages (neutrophil, monocyte, lymphoid, erythroid, megakaryocyte, basophil and mast cell). In keeping with the cell abundance changes, cell fate probability analysis identified increased neutrophil fate probability and decreased erythroid fate probability (Fig. 2A). Interestingly, while the difference in erythroid fate was most marked at the level of MEPs (Fig. 2A), (mean erythroid fate probability 0.46 in STM2457-treated vs 0.56 in Vehicle-treated, *p* = 1.65e−52), it was also detectable in the HSC cluster (Fig. 2A) (mean erythroid probability 0.12 vs 0.15, *p* = 9.54e−86, mean neutrophil probability 0.16 vs 0.12, *p* = 3.25e−62). These results are consistent with a model whereby cell abundance changes induced by isolated catalytic inhibition of METTL3 reflect alterations in cell fate at the level of the least committed progenitors.

To identify potential molecular correlates of altered cell fate, we defined trajectories from HSCs to the seven lineages described above and identified dynamic gene expression along a given trajectory using tradeSeq [10]. Comparing gene expression patterns along the neutrophil trajectory identified 51 genes whose expression dynamics significantly differed in the STM2457-treated cohort (Table S3), including participants in dsRNA and antiviral response (*Ifitm1, Cybb, Oasl2, Ffar2, Cd48*). Among these genes, the neutrophil transcription factor *Cebpd* was upregulated in the STM2457-treated neutrophil trajectory (Fig. 2B) while the monocyte transcription factor *Irf8* was significantly downregulated at the potential branchpoint with the monocytic lineage (Fig. 2B). In the erythroid trajectory 45 genes were identified as dynamically expressed (Table S3) including members of dsRNA-sensing pathways (*Oas2, Oas3, Zbp, Ddx60*) and erythropoietin response genes (*Ccnd2, Isg15, Socs3*).

Of note, cell cycle analysis demonstrated a higher proportion of STM2457-treated cells in G1 phase, indicating an increase in quiescence across the entire HSPC compartment in the STM2457-treated cohort (Figs. 2C and S2C and S3A–O). The inflammatory response gene *Irf7*, upregulated along the erythroid trajectory, (Fig. S2D) has been linked to the regulation of quiescence in stress haematopoiesis [11]. *Ifitm1*, also upregulated along the erythroid trajectory (Figure S2D), is constitutively expressed in HSCs, interferon-induced in erythroid progenitors [12] and is a negative regulator of proliferation [13]. These findings suggest that cell cycle dysregulation may contribute to erythroid progenitor deficiency.

Erythrocytes are the most abundant cells in the body and their generation is critically coupled to cell cycle. The progression of committed erythroid progenitors into terminal differentiation is dependent on progression through S phase [14]. In STM2457-treated samples, early progenitors in the erythroid trajectory were more quiescent, with a significantly higher proportion of cells in G1 phase and a reduction in the fraction of cells progressing into S phase as differentiation proceeds (Figs. 2C and S3I–O). Cell-cycle genes dysregulated in this trajectory included *Hist1h2ap* (Fig. 2D), a nucleosomal core protein whose expression is cell-cycle dependent [15] and *Ccnd2* (Fig. 2D), required for G1/S transition, whose downregulation has been reported to accompany S phase progression and terminal erythroid differentiation [14]. Notably, peripheral blood counts corroborated our observations on the negative impact on erythropoiesis. In the STM2457-treated cohort significantly lower haemoglobin and red blood cell counts (RBC) were accompanied by significantly higher platelets, while total white blood cell counts (WBC) remained largely unaffected (Fig. S2E).

Taken together, our study provides a high-resolution cellular mapping of changes to normal haemopoiesis after catalytic-specific inhibition of the m^6^A writer METTL3 using STM2457. Our results indicate that pharmacological inhibition of METTL3 i) has no impact on the abundance of the least committed progenitors including HSCs, ii) induces gene expression changes consistent with a dsRNA response that is likely to contribute to the observed bias of haematopoiesis towards neutrophil progenitors, with lineage bias and gene expression changes already present in the HSC compartment and iii) causes significant alterations in erythroid differentiation and maturation through aberrant cell cycle regulation. These data provide insights into at least two molecular processes underlying the development of anaemia following isolated catalytic inhibition of METTL3. While we have previously shown that haematopoietic effects induced by STM2457 are transient and rapidly reversible [5], the above observations remain relevant to current high-profile translational efforts with METTL3 small molecule inhibitors currently in clinical development (Phase 1). Whilst our high-resolution data suggest milder, more nuanced and manageable effects of pharmacological METTL3 inhibition on normal haematopoiesis than those observed in METTL3 KO studies, it will nevertheless be important to monitor all haematopoietic parameters in patients treated with METTL3 inhibitors, some of which may potentially act as biomarkers of response.

### Supplementary information

Supplementary Figures

Supplementary Methods

Table S1

Table S2

Table S3

Table S4

## Data Availability

The scRNA-seq datasets generated and analysed during the current study are available under the GEO accession number: GSE228562.
