# Supplementary material for: Pharmacological inhibition of METTL3 impacts specific haematopoietic lineages
Source: Leukemia. 2023 Jul 19;37(10):2133–7. doi: 10.1038/s41375-023-01965-2 (PMC10539174; doi:10.1038/s41375-023-01965-2)
Supplement: Supplementary file 1 — Supplementary Figures [file 41375_2023_1965_MOESM1_ESM.pdf]

Figure S1

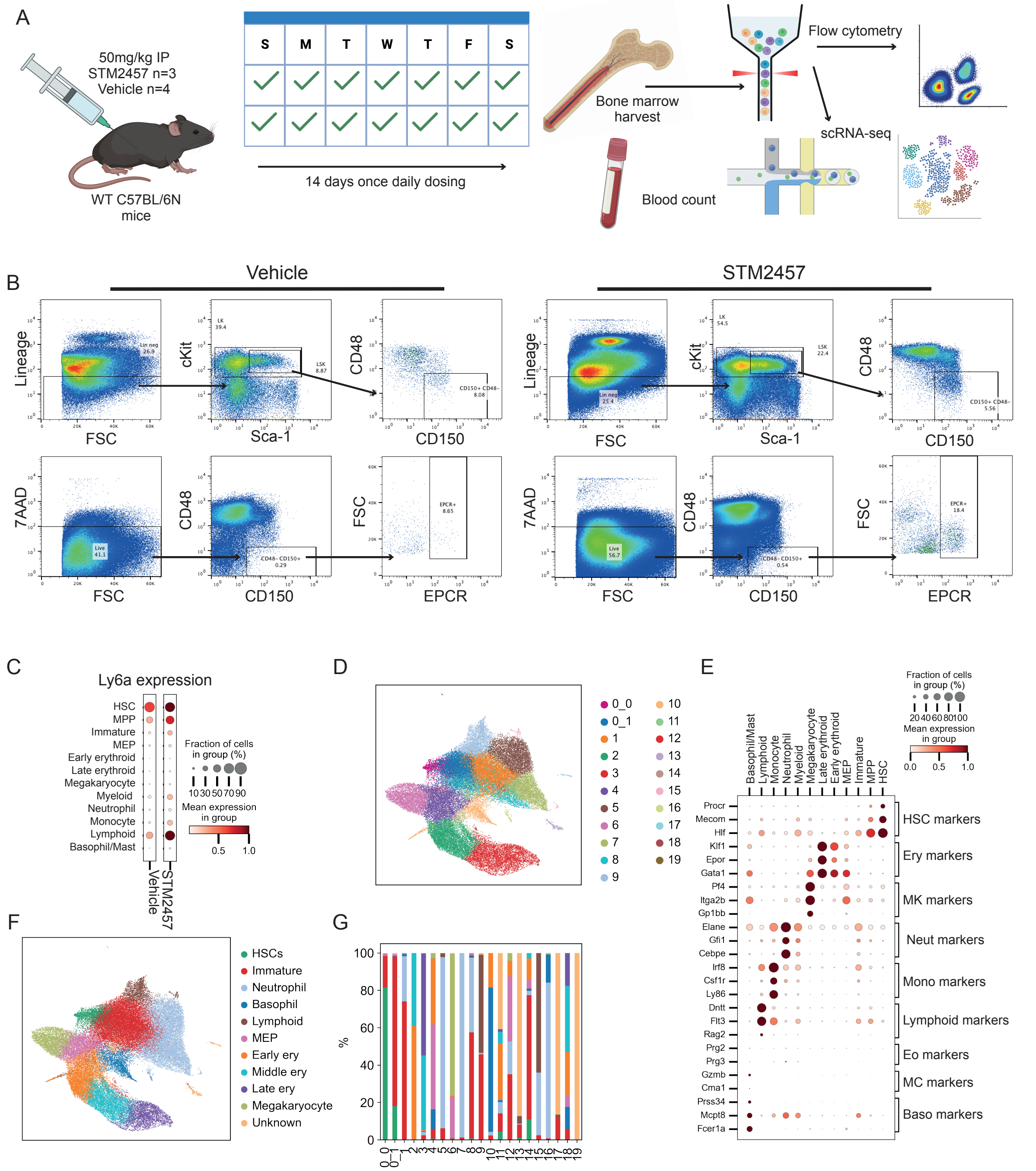


**Figure S1. STM2457 treatment leads to lineage-specific changes in normal bone marrow.**

**A.** Schematic representation of *in vivo* dosing strategy using vehicle or STM2457. **B.** Flow cytometric gating strategy for cell sorting. Representative plots of one vehicle-treated and one STM2457-treated animal are shown. **C.** Ly6a gene expression per cluster. Dots represent

mean log-normalized expression by treatment for each of 12 clusters. Dot size represents the fraction of cells in the cluster which express the gene, colour scale represents mean expression. Mean of 3 STM2457-treated and 4 vehicle-treated animals shown. **D.** UMAP embedding of the whole integrated dataset (52424 cells, STM2457 and vehicle-treated samples) showing 21 Leiden clusters. Clusters containing fewer than 2% of total cells or peripheral clusters representing contaminating mature cell types were excluded from downstream cluster-based analysis. **E.** Cell type-specific marker genes by annotated cluster. Mean log-normalized expression of all seven samples is shown. Dot size represents the fraction of cells in the cluster which express the gene, colour scale represents mean expression. **F.** UMAP embedding of the whole dataset (52424 cells, STM2457 and vehicle-treated samples) showing cell type labels transferred from our previously published reference atlas of 44802 wild-type LK cells. **G.** Label transferred cell identities from the reference dataset represented as a percentage of individual Leiden clusters in the test dataset. Leiden clusters were annotated with cell type identities based on a combination of marker gene expression and the predominant label-transferred cell type in each cluster.

Figure S2

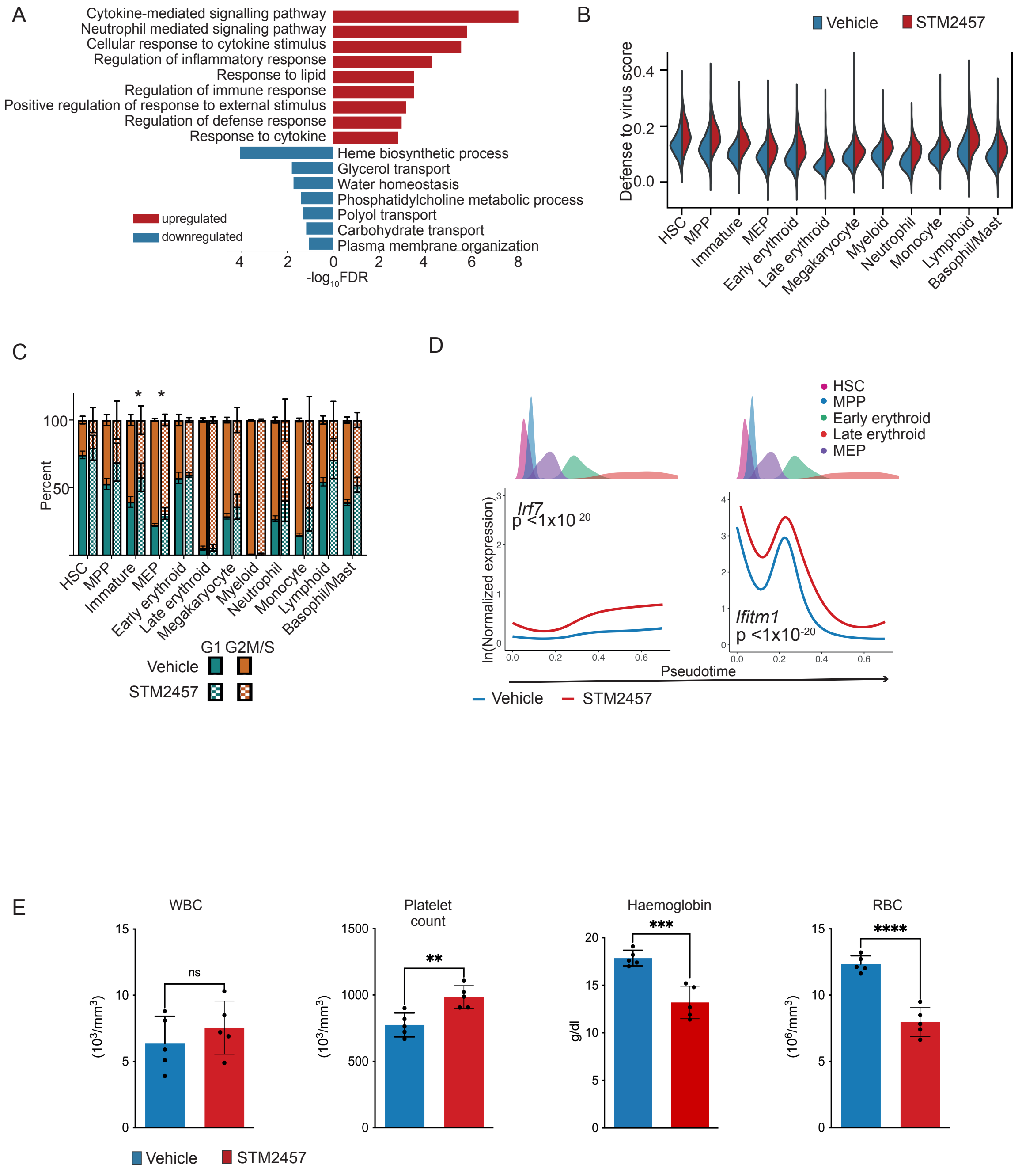


**Figure S2. STM2457 treatment induces gene expression and cell cycle changes with downstream blood count consequences.**

**A.** Gene set enrichment analysis of the list of differentially expressed genes (ranked by adjusted *p* value) between STM2457-treated and vehicle-treated bone marrow. Neutrophil pathways and inflammatory response terms were enriched while heme biosynthesis was

negatively enriched in STM2457-treated samples. **B.** Transcriptional ‘defence to virus score’ computed per cell using genes from GO:0051607. Values are presented per cluster, grouped by treatment. P values were calculated using an independent 2-tailed t-test, corrected for multiple testing using the Benjamini-Hochberg method. The adjusted *p* values are $<1x10^{20}$ for all clusters. **C.** Cell cycle assignment by cluster. Mean and SD of cells in each phase of 3 STM2457-treated and 4 vehicle-treated samples shown. P values were calculated using a two-tailed independent t-test. Asterisks indicate p<0.05. **D.** Differential gene expression dynamics in the erythroid trajectory. The upper panels show the density distribution of cell types along lineage-related pseudotime. The lower panels show gene expression smoothers (line plots) calculated using a generalised additive model for STM2457-treated (red) and vehicle-treated (blue) samples. For p value calculation see materials and methods. **E.** Full blood count results from mice treated with STM2457 and vehicle. White cell count (WBC), platelet count, haemoglobin and red blood cell count are from blood samples taken on day 14 from mice treated daily with either 50mg/kg of STM2457 or vehicle. Mean and SD from 5 STM2457 and 5 vehicle-treated mice are represented. P values were calculated using a two-tailed independent t-test and are indicated as follows: *: p<0.05, **: p<0.01, ***: p<0.005. HSC: Haematopoietic stem cell; MPP: Multipotent progenitor; MEP: Megakaryocyte-erythroid progenitor; WBC: white blood cell count; RBC: Red blood cell count.

Figure S3

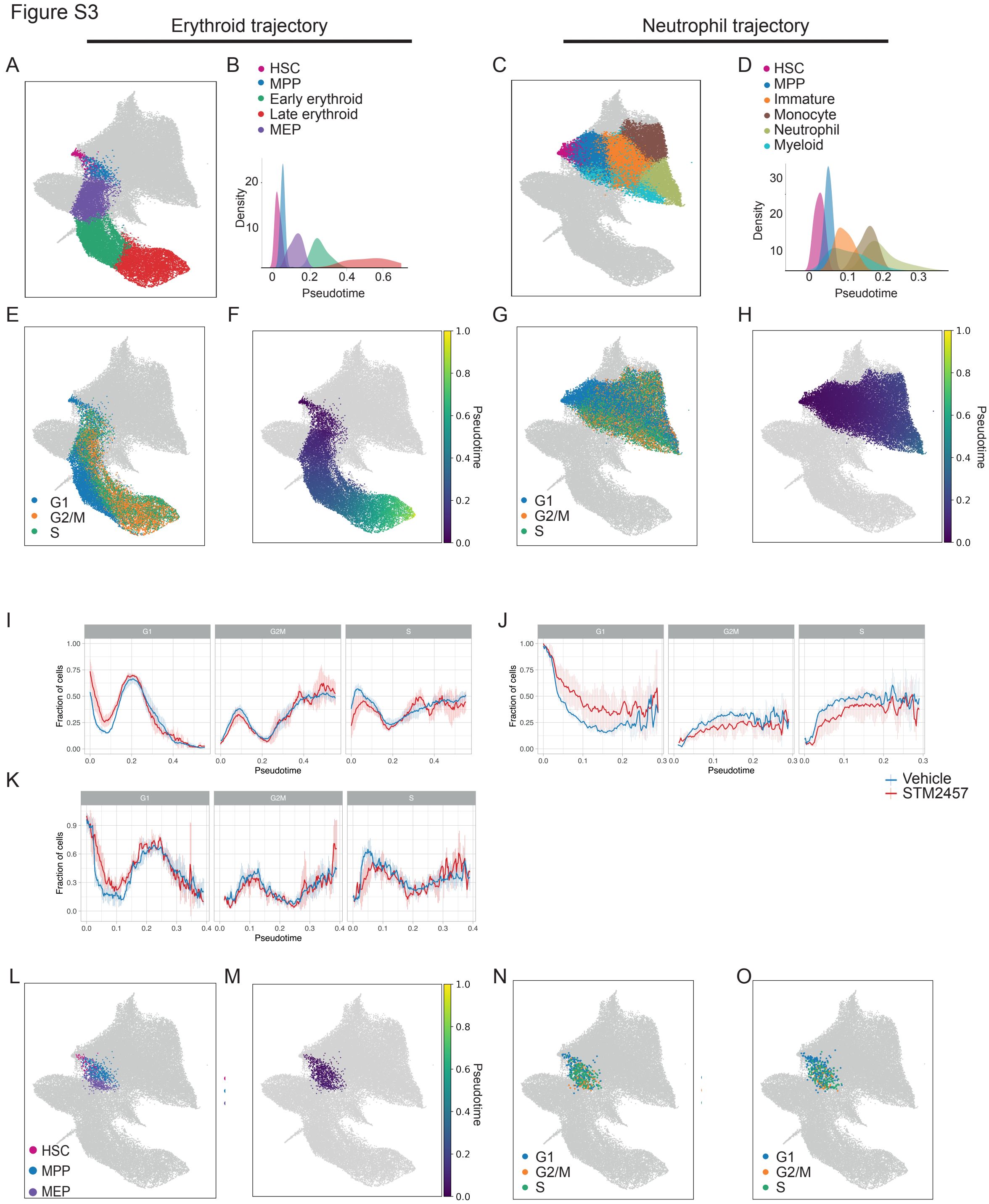


**Figure S3. Small molecule inhibition of METTL3 increases quiescence across the HSPC compartments.**

**A.** Cluster identities of the cells in erythroid trajectory plotted on the UMAP embedding. Lineage trajectories were defined based on CellRank[9] computed cell fate probability (Methods). **B.** Cluster density along pseudotime in the erythroid trajectory. **C.** Cluster identities

of the neutrophil trajectory plotted on the UMAP embedding. Lineage trajectories were defined based on CellRank[9] computed cell fate probability (Methods). **D.** Cluster density along pseudotime in the neutrophil trajectory. **E, F.** Cell cycle phase (E) and pseudotime (F) of the erythroid trajectory plotted on the UMAP embedding. **G, H.** Cell cycle phase (G) and pseudotime (H) of the neutrophil trajectory plotted on the UMAP embedding. **I.** Dynamic cell cycle changes along the erythroid trajectory. Mean (coloured line) and SD (vertical lines) of 3 STM2457-treated and 4 vehicle-treated cohorts are shown. **J.** Dynamic cell cycle changes along the neutrophil trajectory. **K.** Dynamic cell cycle changes in the early erythroid trajectory (pseudotime values <0.4). There is significantly higher G1-phase and a trend towards lower S-phase in the STM2457-treated samples between pseudotime 0.02 and 0.1, corresponding to HSC, MPP and MEP cell types. **L, M.** UMAP embedding showing cells from both treatment cohorts occupying erythroid pseudotime 0.02-0.1. Cell type cluster identity (L) and pseudotime (M) are plotted. **N, O.** Cell cycle phase of vehicle-treated (O) and STM2457-treated (P) erythroid trajectory cells occupying pseudotime 0.02-0.1.
