# Supplementary material for: Pharmacological inhibition of METTL3 impacts specific haematopoietic lineages
Source: Leukemia. 2023 Jul 19;37(10):2133–7. doi: 10.1038/s41375-023-01965-2 (PMC10539174; doi:10.1038/s41375-023-01965-2)
Supplement: Supplementary file 2 — Supplementary Methods [file 41375_2023_1965_MOESM2_ESM.pdf]

Supplementary Methods

Isolation of haematopoietic progenitors

8 to 12-week-old female C57BL/6 mice were treated daily for two weeks with either vehicle or 50 mg/kg STM2457 (STORM). Two weeks post-treatment, bone marrow cells from these mice were freshly dissected and further processed for downstream investigations as described below. All mouse studies were carried out in pathogen-free conditions and in accordance with the Animals (Scientific Procedures) Act 1986, UK and approved by the Ethics Committee at the Sanger Institute. No randomization or blinding performed in this study.

Blood counting

For blood counts, 20 µl of blood was collected from the tail-vein of the mice using a capillary pipette containing anticoagulants (EDTA). The EDTA anti-coagulated blood samples were used to obtain a complete blood count with a Hemavet Mascot Multispecies Hematology System Counter 1500R (CDC Technologies, Inc., Oxford, CT). Samples were counted no longer than 20 minutes after blood was drawn.

Flow cytometric sorting of haematopoietic stem and progenitor cells

Murine bone marrow cells were collected from the femora, tibiae and ilia. Each sample was divided in two and one half processed for scRNA-seq.

Red cells were lysed with ammonium chloride (STEMCELL Technologies, Vancouver, Canada) and haematopoietic progenitors enriched using the EasySep^{TM} Mouse Hematopoietic Progenitor Cell Enrichment Kit (STEMCELL Technologies).

Cells were stained with fluorophore conjugated antibodies c-Kit APC-Cy7 (Biolegend, clone 2B8, 1/200), CD150 PE-Cy7 (Biolegend, clone TC15-12F12.2 1/200), CD48 APC (eBioscience, clone HM48-1, 1/200), EPCR PE (STEMCELL Technologies, clone RMEPCR1560, 1/400), Sca1 BV421 (Biolegend, clone D7, 1/100). The EasySep^{TM} Mouse Hematopoietic Progenitor Cell Isolation Kit (STEMCELL Technologies) and BV510 streptavidin (Biolegend) was used to stain lineage-positive cells. Dead cells were excluded using 7-aminoactinomycin D (Thermo Fisher Scientific). An aliquot of non-lineage- depleted cells was retained for unstained and single-stain controls.

Cells were analysed and sorted on an Influx sorter (BD Biosciences); 20,000 Lineage-negative, c-kit + cells per mouse were sorted into PBS + 2% FCS for scRNA-seq analysis (10x Genomics, Pleasanton, CA). Cells were then processed according to manufacturer's protocol using Chromium Single Cell 3' GEM, Library & Gel Bead Kit v3.1 reagents and sequenced on the Illumina Novaseq 6000 platform.

scRNA-seq analysis

Raw reads were mapped to the mm10 genome and pre-processing was performed using the CellRanger pipeline (v 6.0.1) using default parameters.

Subsequent analysis was performed in Scanpy(1). Cells expressing fewer than 1,200 genes or with over 5% of reads mapping to mitochondrial genes were excluded. Scrublet(2) was used to exclude potential doublets. Of the four STM2457-treated mice, one outlier sample with significantly lower read counts and gene counts could not be integrated into a shared manifold due to strong batch effect which could not be corrected. This sample was therefore not considered in downstream analysis. A total

of 52,424 transcriptomes passed these quality controls from 4 vehicle-treated (n= 8,113, 7,585, 6,756, 6,656) and 3 STM2457-treated (n= 7,931, 7,731, 7,652) animals and were taken forward for subsequent analysis in an integrated manifold comprising all samples from vehicle and STM2457-treated animals.

Counts were log-normalized and 1,105 highly variable genes (HVG) were selected following the method of Satija et al(3) implemented through Scanpy. Counts were scaled and cells scored and allocated to a cell cycle phase by calculating the difference in mean expression between a randomly sampled reference gene set and the given list of cell cycle genes (https://github.com/theislab/scanpy_usage/blob/master/180209_cell_cycle/data/regev_lab _cell_cycle_genes.txt). The effect of cell cycle was regressed out to reduce impact on visualisation and clustering.

Cell-cycle regressed values of 1,105 highly variable genes were used to compute 50 principal components. Correction for the batch effect of sample identity was performed using Harmony(4) and the top 30 Harmony batch-corrected principal components were utilised to calculate a k-nearest neighbour graph with k=15 and a Euclidean distance metric. This was then taken as inputs to visualise the data as a UMAP embedding(5). Data were clustered using the Leiden algorithm in Scanpy, following the implementation of Traag(6).

The integrated dataset was mapped onto a previously published reference wild-type droplet-based scRNA-seq dataset of 44,802 mouse lineage negative c-kit+ (LK) cells(7) as previously described(8). Marker gene expression and projection of the dataset onto a reference LK dataset allowed cell type annotation of the 20 clusters (Figure 1C, Suppl Fig1 D-G). Peripheral clusters containing contaminating mature cell types, and clusters containing fewer than 1,000 cells (<2% of total) were not considered in downstream cluster analysis.

As a surrogate of effective METTL3 inhibition, the Scanpy command sc.tl.score_genes() was used to compute a gene score per cell for genes included in the GO term GO:GO:0051607 (defence to virus). Analogous to the calculation of cell cycle score, the gene score calculates the mean expression difference between a given gene list and a randomly sampled gene list.

Pseudobulk differential expression analysis

A pseudobulk method(9) was employed to perform differential gene expression analysis for the dataset as a whole and for individual clusters(10). Low count genes were excluded on a per-sample or per-cluster basis, retaining only genes with an estimated expression above 1 transcript per million in more than 25% of cells(11). Differential expression between treatment conditions was computed using the likelihood ratio test in EdgeR(12,13) with experimental batch used as a covariate in the model. Genes with log2 fold changes >0.5 or <-0.5 and Benjamini-Hochberg (BH) adjusted p values <0.05 were considered significant. Gene set enrichment analysis using gene lists ordered by BH-adjusted p value were used as input for gene set enrichment analysis in EnrichR(14).

Differential cell abundance analysis

Treatment-related differences in cellular abundance were computed using the python package MELD(15). First, a cell-similarity graph was calculated based on Euclidean distance in the shared PCA space of the integrated dataset. Then for each sample a kernel density estimate (KDE) was computed and smoothed over the cell similarity graph with 8 nearest neighbours and smoothing parameter beta = 15. Differential

abundance was then calculated as the relative likelihood of observing a cell in either the STM2457- or vehicle-treated condition following cell-wise L1 normalization of the sample KDEs. The mean of relative likelihoods from all pairwise comparisons was used to plot differential abundance on the UMAP embedding (Figure 1D), while comparison of the KDEs between treatments within experimental batch permitted calculation of statistical significance. An independent t-test was used to calculate significance, corrected for multiple testing with the Benjamini-Hochberg method. Differential abundance with a BH-corrected p value <0.2 was considered significant.

Cell fate probability analysis
Cell fate probability was computed using the python package CellRank(16). Root cells were assigned by calculating the HSC score(17) to infer the cells with the transcriptome most highly enriched for long term repopulating HSC genes. Diffusion pseudotime values were calculated using the scanpy function 'scanpy.tl.dpt' with default settings. For each sample a single-cell transition matrix was computed using the CellRank pseudotime kernel and 'soft' threshold scheme. Terminally differentiated macrostates were identified using the Generalized Perron Cluster Cluster Analysis (GPCCA) estimator, and absorption (fate) probabilities towards these states were then computed. These inferred fate probabilities were then compared for each cluster between treatment conditions using the Seurat 'find markers' function(18) to perform logistic regression and a likelihood ratio test. Experimental batch was included in the logistic regression model. Cell fate probability differences were considered significant if Benjamini-Hochberg adjusted p values were <0.05.

Trajectory-based differential gene expression dynamics
To identify potential gene-expression correlates of altered cell fate, gene expression dynamics were compared along pseudotime using the R package tradeSeq(19). Using cell fate probabilities computed previously, cells were assigned to seven trajectories (Erythroid, Neutrophil, Monocyte, Megakaryocyte, Lymphoid, Basophil and Mast Cell). Each cell was assigned to a trajectory if its cell fate probability for that trajectory was either the maximum or 0.6 times the maximum fate value. This permitted cells to be assigned to more than one trajectory at lower pseudotime values, indicating a more immature progenitor state. The erythroid and neutrophil trajectories were then selected for downstream analysis. To exclude a small number of outliers, cells were excluded from the erythroid trajectory if they were not also assigned to the HSC, MEP or early/late erythroid clusters, and excluded from the neutrophil clusters if they were not also assigned to the HSC, immature, myeloid, neutrophil or monocyte clusters. To ensure sufficient representation along pseudotime in the erythroid trajectory, the pseudotime range 0-0.6 was included.
TradeSeq was then used to fit a negative binomial generalized additive model (fitGAM function, 6 knots) with experimental batch included as a covariate in the model. Expression patterns along lineage-specific pseudotime were then compared between treatment conditions using the 'condition test' function which applies a Wald test to the parameters of the fitted model to test a null hypothesis that these parameters do not differ between the two conditions. Genes with log2 fold changes of >1.0 and Benjamini-Hochberg adjusted p values <0.05 were considered significant.

RNA nucleoside quantification by mass spectrometry
8 to 12-week-old female C57BL/6 mice were treated daily for two weeks with either vehicle or 50 mg/kg STM2457 (STORM) and whole bone marrow from these mice was

harvested. Subsequent total RNA extraction from cells of all origins was performed using QIAZOL Reagents (QIAGEN), following the manufacturer's protocol. Nucleosides were prepared from total RNA by addition of nuclease digest mix. Each 100 µL volume contained 62.5 units of Benzonase (Sigma Aldrich), 5 units of Antarctic Phosphatase (NEB) and 10 mU/µL of Phosphodiesterase I (PDEI) from *Crotalus adamanteus* venom (Sigma Aldrich) made up in a buffer composed of 20 mM Tris-HCl (pH 8), 20 mM $MgCl_2$ and 100 mM NaCl. Nucleosides were liberated by digestion at 37ºC overnight. The following morning the sample was cooled to room temperature and 100 µl of ice cold 2x Mass Spec buffer was added (0.1% formic acid containing internal standard).

Nucleosides were then quantified by LC-MS using a Sciex 4500 triple quadrupole mass spectrometer attached to either a U3000 or 1290 liquid chromatograph (Thermo Scientific or Agilent, respectively). Nucleosides were separated across a HSS T3 column (2mm x 10mm with 1.8 µm particles, Waters) using an increasing gradient of 2-15% mobile phase B. The mobile phases were 0.1% v/v formic acid in water or acetonitrile for mobile phase A and B, respectively, and the flow was held at 300 µL/min. Nucleoside concentrations were extrapolated from a concentration curve of external standards and expressed as modified nucleoside relative to the total amount of canonical nucleosides. All data in this section were plotted using GraphPad Prism (Version 9).

Statistics and visualisation

Statistical analysis was performed using the python package statsmodels. Unless otherwise specified, t-tests were independent student's t-tests. Homogeneity of variance was first determined using Levene's and Bartlett's tests. Analysis and visualisation were also performed in GraphPad Prism. Visualisations were generated using Matplotlib, Seaborn and ggplot2.

Data accessibility

The scRNA-seq datasets generated and analysed during the current study are available under the GEO accession number: GSE228562.

References

1. Wolf FA, Angerer P, Theis FJ. SCANPY: large-scale single-cell gene expression data analysis. Genome Biology. 2018 Feb 6;19(1):15.

2. Wolock SL, Lopez R, Klein AM. Scrublet: Computational Identification of Cell Doublets in Single-Cell Transcriptomic Data. Cell Systems. 2019 Apr;8(4):281-291.e9.

3. Satija R, Farrell JA, Gennert D, Schier AF, Regev A. Spatial reconstruction of single-cell gene expression data. Nat Biotechnol. 2015 May;33(5):495–502.

4. Korsunsky I, Millard N, Fan J, Slowikowski K, Zhang F, Wei K, et al. Fast, sensitive and accurate integration of single-cell data with Harmony. Nature Methods. 2019 Dec;16(12):1289–96.

5. McInnes L, Healy J, Melville J. UMAP: Uniform Manifold Approximation and Projection for Dimension Reduction [Internet]. arXiv; 2020 [cited 2023 Mar 28]. Available from: http://arxiv.org/abs/1802.03426

6. Traag VA, Waltman L, van Eck NJ. From Louvain to Leiden: guaranteeing well-connected communities. Scientific Reports. 2019 Mar 26;9(1):5233.

7. Dahlin JS, Hamey FK, Pijuan-Sala B, Shepherd M, Lau WWY, Nestorowa S, et al. A single-cell hematopoietic landscape resolves 8 lineage trajectories and defects in Kit mutant mice. Blood. 2018 May 24;131(21):e1–11.

8. Li J, Williams MJ, Park HJ, Bastos HP, Wang X, Prins D, et al. STAT1 is essential for HSC function and maintains MHCIIhi stem cells that resist myeloablation and neoplastic expansion. Blood. 2022 Oct 6;140(14):1592–606.

9. Lun ATL, Marioni JC. Overcoming confounding plate effects in differential expression analyses of single-cell RNA-seq data. Biostatistics. 2017 Jul 1;18(3):451–64.

10. Lenaerts A, Kucinski I, Deboutte W, Derecka M, Cauchy P, Manke T, et al. EBF1 primes B-lymphoid enhancers and limits the myeloid bias in murine multipotent progenitors. Journal of Experimental Medicine. 2022 Sep 1;219(11):e20212437.

11. Soneson C, Robinson MD. Bias, robustness and scalability in single-cell differential expression analysis. Nat Methods. 2018 Apr;15(4):255–61.

12. Robinson MD, McCarthy DJ, Smyth GK. edgeR : a Bioconductor package for differential expression analysis of digital gene expression data. Bioinformatics. 2010 Jan 1;26(1):139–40.

13. McCarthy DJ, Chen Y, Smyth GK. Differential expression analysis of multifactor RNA-Seq experiments with respect to biological variation. Nucleic Acids Research. 2012 May 1;40(10):4288–97.

14. Kuleshov MV, Jones MR, Rouillard AD, Fernandez NF, Duan Q, Wang Z, et al. Enrichr: a comprehensive gene set enrichment analysis web server 2016 update. Nucleic Acids Res. 2016 Jul 8;44(W1):W90–7.

15. Burkhardt DB, Stanley JS, Tong A, Perdigoto AL, Gigante SA, Herold KC, et al. Quantifying the effect of experimental perturbations at single-cell resolution. Nat Biotechnol. 2021 May;39(5):619–29.

16. Lange M, Bergen V, Klein M, Setty M, Reuter B, Bakhti M, et al. CellRank for directed single-cell fate mapping. Nat Methods. 2022 Feb;19(2):159–70.

17. Hamey FK, Göttgens B. Machine learning predicts putative hematopoietic stem cells within large single-cell transcriptomics data sets. Experimental Hematology. 2019 Oct;78:11–20.

18. Hao Y, Hao S, Andersen-Nissen E, Mauck WM, Zheng S, Butler A, et al. Integrated analysis of multimodal single-cell data. Cell. 2021 Jun 24;184(13):3573-3587.e29.

19. Van den Berge K, Roux de Bézieux H, Street K, Saelens W, Cannoodt R, Saeys Y, et al. Trajectory-based differential expression analysis for single-cell sequencing data. Nat Commun. 2020 Mar 5;11(1):1201.
